# Tecovirimat for the treatment of severe Mpox in Germany

**DOI:** 10.1007/s15010-023-02049-0

**Published:** 2023-06-05

**Authors:** Lennart Hermanussen, Thomas Theo Brehm, Timo Wolf, Christoph Boesecke, Stefan Schlabe, Frauke Borgans, Malte B. Monin, Björn-Erik Ole Jensen, Stefan Windhaber, Stefan Scholten, Sabine Jordan, Marc Lütgehetmann, Julian Schulze zur Wiesch, Marylyn M. Addo, Agata Mikolajewska, Michaela Niebank, Stefan Schmiedel

**Affiliations:** 1 Division of Infectious Diseases, I. Department of Internal Medicine, University Medical Center Hamburg-Eppendorf, Martinistraße 52, 20246 Hamburg, Germany; 2 German Center for Infection Research (DZIF), Partner-Site Hamburg-Lübeck-Borstel-Riems, Hamburg, Germany; 3 Internal Medicine II, Department of Infectious Diseases, University Hospital, Frankfurt, Germany; 4 Department of Medicine I, Bonn University Hospital, Bonn, Germany; 5 German Center for Infection Research (DZIF), Partner-Site Cologne-Bonn, Bonn, Germany; 6 Department of Gastroenterology, Hepatology and Infectious Diseases, Medical Faculty, Düsseldorf University Hospital, Heinrich-Heine-University, Düsseldorf, Germany; 7 Praxis Hohenstaufenring in den RingColonnaden, Cologne, Germany; 8 Institute of Medical Microbiology, Virology and Hygiene, University Medical Center Hamburg-Eppendorf, Hamburg, Germany; 9 Institute for Infection Research and Vaccine Development (IIRVD), University Medical Center Hamburg-Eppendorf, Hamburg, Germany; 10 Centre for Biological Threats and Special Pathogens (ZBS), Robert Koch Institute, Berlin, Germany

**Keywords:** Mpox, Germany, Tecovirimat, HIV, MSM

## Abstract

**Background:**

In May 2022, a multi-national mpox outbreak was reported in several non-endemic countries. The only licensed treatment for mpox in the European Union is the orally available small molecule tecovirimat, which in *Orthopox* viruses inhibits the function of a major envelope protein required for the production of extracellular virus.

**Methods:**

We identified presumably all patients with mpox that were treated with tecovirimat in Germany between the onset of the outbreak in May 2022 and March 2023 and obtained demographic and clinical characteristics by standardized case report forms.

**Results:**

A total of twelve patients with mpox were treated with tecovirimat in Germany in the study period. All but one patient identified as men who have sex with men (MSM) who were most likely infected with mpox virus (MPXV) through sexual contact. Eight of them were people living with HIV (PLWH), one of whom was newly diagnosed with HIV at the time of mpox, and four had CD4+ counts below 200/µl. Criteria for treatment with tecovirimat included severe immunosuppression, severe generalized and/or protracted symptoms, a high or increasing number of lesions, and the type and location of lesions (e.g., facial or oral soft tissue involvement, imminent epiglottitis, or tonsillar swelling). Patients were treated with tecovirimat for between six and 28 days. Therapy was generally well-tolerated, and all patients showed clinical resolution.

**Conclusions:**

In this cohort of twelve patients with severe mpox, treatment with tecovirimat was well tolerated and all individuals showed clinical improvement.

## Introduction

Mpox is a viral zoonotic disease that was first reported in 1970 in the Democratic Republic of Congo [1]. In the past decades, the virus has caused regular outbreaks in certain regions in Central and West Africa [2]. Imported cases have been rare events associated with travel to endemic regions or contact with infected imported rodents [3–7]. In May 2022, a multi-national mpox outbreak was first reported in several non-endemic countries, predominantly affecting men who have sex with men (MSM) [8, 9]. On July 23, 2022, mpox was escalated by the World Health Organization (WHO) to a Public Health Emergency of International Concern (PHEIC) [10]. As of March 16, 2023, 86,496 cases including 111 fatalities have been registered by the WHO from 110 countries [11]. Among them were 3,692 cases from Germany, from where no mpox-related deaths have been reported. The only licensed treatment for mpox in the European Union is the oral small molecule tecovirimat (TPOXX®), which in *Orthopox* viruses inhibits the function of a major envelope protein required for the production of extracellular virus. Tecovirimat was authorized by the European Medicines Agency (EMA) for the treatment of smallpox, mpox and cowpox in adults and children with a body weight of at least 13 kg in January 2022 [12]. A human safety study did not show any severe side effects in immunocompetent individuals [13]. Data from ongoing clinical trials are not yet accessible, however, some data on therapeutic use in humans is available from observational studies [14–17]. Here we describe the first twelve consecutive mpox cases that were treated with tecovirimat in Germany since the onset of the current outbreak.

## Materials and methods

We aimed to identify all patients with mpox that were treated with tecovirimat in Germany between the onset of the outbreak in May 2022 and March 2023. Each treatment indication was consensually discussed in the STAKOB (Permanent Working Group of Competence and Treatment Centers for High-consequence Infectious Diseases at Robert Koch Institute) (www.rki.de/stakob). A standardized case report form was used to obtain the demographic and clinical characteristics of patients from the treating physicians.

Molecular diagnostics were performed as previously established and published [18, 19].

## Results

Between May 2022 and March 2023, twelve patients with confirmed mpox were treated with tecovirimat in Germany (Table 1). Five cases are already published as a case series (ID 1–3), or clinical images (ID 3–5) [20–23]. All patients were male. All but one patient identified as MSM and mpox virus (MPXV) was most likely transmitted through sexual contact. One patient did not identify as MSM and denied any prolonged skin-to-skin contact with other individuals, so the route of transmission in this patient remains unknown. Eight patients (67%) had previously (*n* = 7) or at the time of presentation (*n* = 1) been diagnosed with human immune deficiency virus (HIV) infection. Among these patients, four had CD4+ counts below 200/µl and two of them had HIV viremia as they did not receive anti-retroviral therapy (ART) at the time of MPXV infection. One patient was vaccinated against smallpox in his childhood and against mpox three weeks before the detection of MPXV infection (ID 12). Another patient was vaccinated against mpox just 4 days before the confirmed infection (ID 2). Accordingly, the majority of patients (*n* = 10, 83%) had a negative vaccination status for mpox.

**Table 1:**
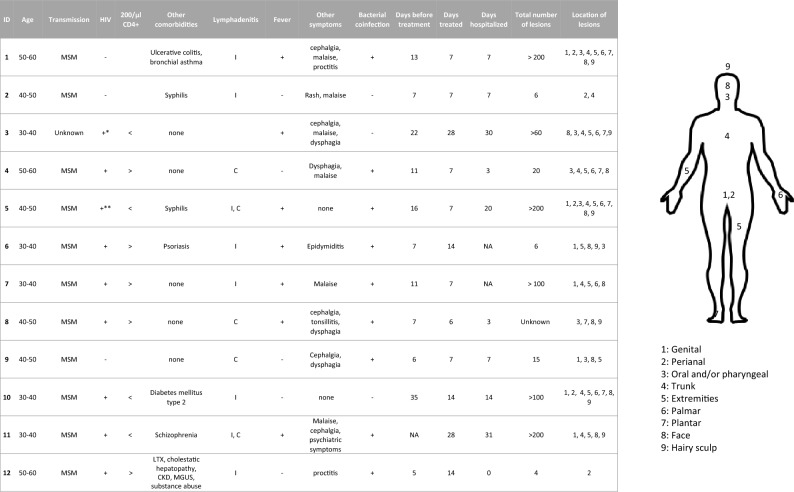
Patient characteristics

All patients were male. All patients suffered from lymphadenitis, inguinal (I), cervical (C), and/or popliteal (P). The body scheme indicates the location of lesions. HIV human immunodeficiency virus, LTX liver transplantation, MGUS monoclonal gammopathy of undetermined significance, CKD chronic kidney disease

*HIV viremia

**HIV viremia, current diagnosis

MPXV was detected in skin lesions of all patients, in all five tested blood samples, and in all six tested nasopharyngeal swabs by RT-PCR. In two cases there was a prolonged regression of lesions in which MPXV could still be detected for three months (ID 10), or at least for two months before a loss to follow-up (ID 3). Three patients presented with more than 200 skin lesions, three patients with between 50 and 100 lesions, and six patients with less than 50 lesions. In one case the number of lesions was not documented. Lesions were most commonly located in the penile and scrotal (*n* = 6, 50%), facial (*n* = 6, 50%), oral (*n* = 5, 42%), as well as perianal and gluteal (*n* = 3, 25%) regions. A total of six (50%) patients showed generalized involvement with lesions on the entire integument. All patients suffered from cervical and/or inguinal lymphadenopathy. Other commonly reported symptoms were fever (*n* = 7, 58%, malaise (*n* = 7, 58%), cephalgia (*n* = 5, 42%), and dysphagia (*n* = 3, 25%). Mild proctitis was seen in a patient with known ulcerative colitis, who was in sustained clinical remission since being treated with vedolizumab (ID 1), and in a person living with HIV (PLWH) who had received a liver transplant (ID 12) (*n* = 2, 17%). Five patients (42%) reported severe pains of lesions or lymphadenitis and had temporarily been treated with analgesic opioids. Additional antibiotic treatment was performed in ten patients (83%) for suspected bacterial coinfection. Cultural evidence for bacteria could only be obtained in one patient by a genital swab. In this case, a mixed flora of gram-negative pathogens has grown. Two patients were simultaneously diagnosed with acute syphilis.

All but one patient (*n* = 11, 92%) were hospitalized, the median duration of hospitalization was seven days (IQR 3–31). The most common reasons for hospital admission were suspected bacterial coinfection with an indication for i.v. antibiotic therapy, severe pain of the lesions, and critical locations at risk of airway obstruction.

Treatment with tecovirimat was initiated in this cohort if one or more of the following criteria were met:

- severe immunosuppression due to HIV infection and a low CD4+ count (*n* = 4, 33%)
- severe immunosuppression after solid organ transplantation (*n* = 1, 8%)
- severe generalized and/or protracted symptoms (*n* = 4, 33%)
- a high or increasing number of lesions (> 50–100) (*n* = 7, 58%)
- type and location of lesions (e.g., facial or enoral soft tissue involvement, imminent epiglottitis, or tonsillar swelling) (*n* = 6, 50%)

Those conditions were considered potential treatment indications on an individual patient basis. In all cases, physicians assumed active disease manifested by severe, generalized symptoms or by constant recurrence of lesions. Treatment with tecovirimat was initiated after a median of eleven days (IQR 5–35) after symptom onset. Notably, one patient (ID 10) was symptomatic with newly occurring lesions over five weeks and only recovered after treatment initiation with tecovirimat, 35 days after symptom onset. Tecovirimat was administered orally to all patients at a dose of 600 mg bid. The treatment duration was based on clinical response: one patient was treated for 6 days, seven patients for 7 days, one patient for 14 days, and two patients for 28 days. Treatment was well tolerated, no severe side effects were supported. Only one patient (ID 3) showed a transient increase in transaminase levels, that normalized under ongoing tecovirimat treatment. For four patients longitudinal virological data were available (ID 1–3, ID 11). In blood samples, the viral load decreased upon treatment, as previously published [20].

The two patients treated for 28 days had an HIV infection with low CD4+ cell counts. The first patient who was treated for 28 days showed clinical and virological relapse aight days after the initial improvement of his clinical condition and discharge from the hospital (ID 3). He was readmitted with malaise, severe right leg pain and swelling, popliteal and inguinal lymphadenopathy, and newly developed lesions. When he was first discharged, a low viral load of MPXV was still detected in blood samples (CT 30,5). Within the tecovirimat therapy-free interval, the viral load of MPXV had increased again (CT 26,5). Magnetic resonance imaging (MRI) revealed infectious myositis with an abscess formation in the gastrocnemius muscle [23]. Specimen collection revealed MPXV in high concentrations, with no evidence of any other infectious etiology. Treatment with tecovirimat was restarted, and the patient did not receive any additional antibiotics. His clinical condition started improving in the first week of the renewed 14 days of therapy.

**Fig. 1:**
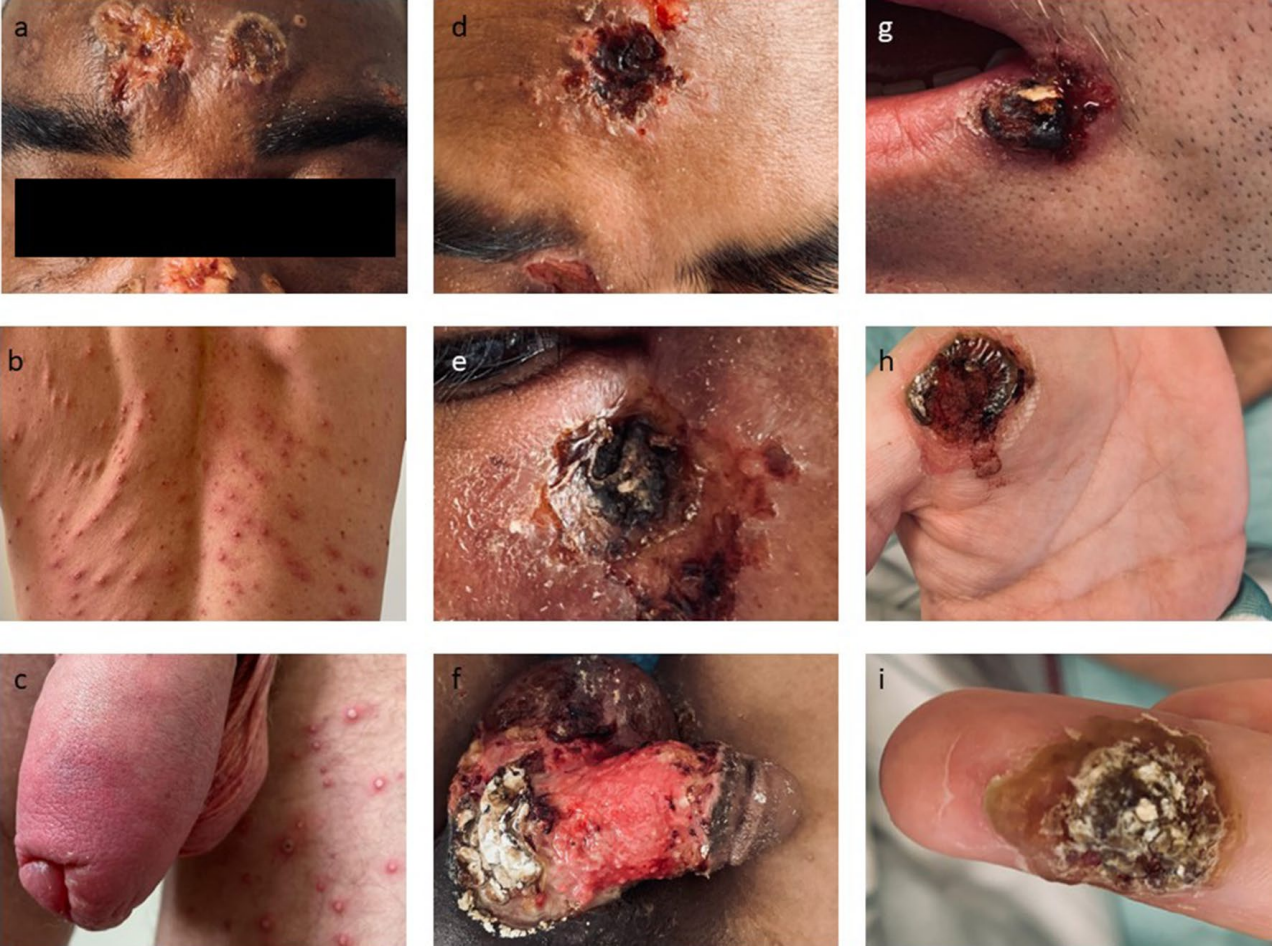
Exemplary photos of lesions in patient ID 3 (**a**), ID 7 (**b**, **c**), ID 10 (**g**, **h**, **i**), and ID 11 (**d**, **e**, **f**)

The other patient did not show adequate clinical recovery after 14 days of treatment: he still showed extended and painful lesions as well as MPXV viremia (CT 32,5) (ID 11). Therefore, and also based on the experience with patient ID 3, the duration of treatment was extended. At the time of admission, he showed psychiatric symptoms and upon in-hospital treatment with tecovirimat, he developed a psychosis. According to his medical history, a similar episode had occurred several years ago, so that he had been diagnosed with schizophrenia. However, the patient was free of psychiatric symptoms for years even without antipsychotic therapy. Mpox encephalitis was suspected based on the detection of MPXV in cerebrospinal fluid. However, cranial MRI showed normal cerebral findings. Upon prolonged tecovirimat and antipsychotic therapy, the condition constantly improved and at the time of discharge no MPXV viremia was detected (Fig. 1).

## Discussion

Here we present demographic and clinical data of the twelve mpox patients who were considered severe courses of mpox and were treated with tecovirimat in Germany between May 2022 and March 2023. Due to low stockpiles of tecovirimat, and the possible rapid development of viral resistance in in vitro cell culture, in Germany, the use of this agent is currently limited to severe disease courses. While we cannot exclude that other severe mpox courses occurred that are not part of this case series, there are no known fatalities from mpox in Germany [11]. In our cohort, mpox was associated with high morbidity and prolonged hospital stays of up to more than 30 days.

All patients in our case series tolerated tecovirimat treatment well. As mpox is generally considered a self-limiting disease, we are not able to reliably determine whether the treatment had contributed to clinical recovery. One patient (ID 3) showed clinical relapse after ending tecovirimat therapy after 14 days and rapid improvement in the second treatment cycle. Also, for another patient (ID 11) the therapy cycle needed to be extended to a total of 28 days. Of note, these two patients showed a constant decline of MPXV viremia as long as treated, giving no indication of viral resistance. These patients were among four PLWH with low CD4+ counts [21]. This suggests a relevant role of the adaptive CD4+ cell response in the viral control of MPXV infection and is in line with a recent global case series reporting on mpox in PLWH with low CD4+ T cells [24]. As previously published, the severe mpox course in a patient treated with vedolizumab for ulcerative colitis might be coincidental (ID 1) [20]. However, vedolizumab is a recombinant antibody targeting the α4β7 integrin that aides lymphocytes and especially CD4+ T cell homing to the lamina propria from gut-inductive sites where immune responses are classically first induced (Peyer's patches and mesenteric lymph nodes) [25]. Antigen-specific α4β7 + CD4+ T cells are also found in genital mucosa [26–28] and blockade of this integrin by vedolizumab might impact the local adaptive T cell response against MPXV [20]. For this patient, no other potential risk factors for a severe mpox course could be identified.

Two patients were simultaneously diagnosed with acute syphilis. The potential impact of syphilis or other sexually transmitted infections (STIs) on a more severe course of mpox or on an individual`s predisposition for MPXV infection is not known. Providers are advised to diligently rule out any additional STI when assessing a possible mpox patient without delaying a potential tecovirimat therapy.

Many patients in this series were additionally treated with antibiotics for suspected bacterial coinfection. However, it is challenging to distinguish MPXV infection alone from bacterial superinfection, as mpox itself can cause high inflammatory parameters and even be responsible for a muscle abscess in one case [20, 23].

Besides patient characteristics like relevant immunosuppression, also progressive development of skin lesions and persistently high MPXV viremia were reasons for tecovirimat treatment initiation. The detection of high MPXV concentrations in the blood seems to be associated with severe clinical courses [20]—comparable to the virological findings in patients with other viral infections like SARS-CoV-2 [29, 30]]. In this case series, all tested blood samples were positive for MPXV.

In one patient MPXV was persistently detected after 3 months (ID 10), and in another patient for at least 2 months before loss to follow-up (ID 3). Both patients showed residual lesions despite the resolution of other symptoms.

Further studies need to evaluate the safety, tolerability, and efficacy of tecovirimat in early and delayed treatment initiation, the duration of therapy, and response-guided treatment algorithms. Additionally, virologic and laboratory tools need to be established to assess the treatment response, infectivity, and early detection of possible escape mutations.

## Conclusion

Between May 2022 and February 2023, twelve patients with severe mpox were treated with tecovirimat in Germany. Treatment indications were severe immunosuppression due to HIV infection with low CD4+ T cell counts (< 200/µl) and severity or location of lesions. While tecovirimat was well tolerated and all patients showed clinical recovery, treatment response can only be reliably assessed by prospective studies.

## Data Availability

The original data and materials are available from the corresponding author, SS, upon reasonable request.
